# Modification of the association between recreational physical activity and survival after breast cancer by promoter methylation in breast cancer-related genes

**DOI:** 10.1186/s13058-017-0811-z

**Published:** 2017-02-21

**Authors:** Lauren E. McCullough, Jia Chen, Yoon Hee Cho, Nikhil K. Khankari, Patrick T. Bradshaw, Alexandra J. White, Susan L. Teitelbaum, Mary Beth Terry, Alfred I. Neugut, Hanina Hibshoosh, Regina M. Santella, Marilie D. Gammon

**Affiliations:** 1 Department of Epidemiology, Emory University, Atlanta, GA 30322 USA; 2 Department of Preventive Medicine, Icahn School of Medicine at Mount Sinai, New York, NY 10029 USA; 3 Department of Pediatrics, Icahn School of Medicine at Mount Sinai, New York, NY 10029 USA; 4 Department of Oncological Science, Icahn School of Medicine at Mount Sinai, New York, NY 10029 USA; 5 Department of Biomedical and Pharmaceutical Sciences, University of Montana, Missoula, MT 59812 USA; 6 Division of Epidemiology, Vanderbilt University Medical Center, Nashville, TN 37203 USA; 7 Division of Epidemiology, University of California Berkeley, Berkeley, CA 94720 USA; 8 Epidemiology Branch, National Institute of Environmental Health Science, Research Triangle Park, NC 27709 USA; 9 Department of Epidemiology, Columbia University, New York, NY 10032 USA; 10 Department of Medicine, Columbia University, New York, NY 10032 USA; 11 Department of Pathology, Columbia University, New York, NY 10032 USA; 12 Department of Environmental Health Sciences, Columbia University, New York, NY 10032 USA; 13 Department of Epidemiology, University of North Carolina at Chapel Hill, Chapel Hill, NC 27599 USA

**Keywords:** Physical activity, Epigenetics, Methylation, Breast cancer, Survival

## Abstract

**Background:**

Mechanisms underlying the inverse association between physical activity and survival after breast cancer are unresolved, but DNA methylation may play a role. We hypothesized that promoter methylation of breast cancer-related genes, as well as global methylation, may modify the association between prediagnostic recreational physical activity (RPA) and breast cancer mortality.

**Methods:**

Using a population-based sample of 1254 women diagnosed with first primary breast cancer, we examined modification of the RPA-mortality association by gene-specific promoter methylation and global methylation. Average lifetime RPA was assessed from menarche to diagnosis through structured in-home interviews. Promoter methylation of 13 breast cancer-related genes was evaluated in archived tumor by methylation-specific polymerase chain reaction and MethyLight assay. Global methylation in white blood cell DNA was determined at long interspersed nucleotide element 1 and by the luminometric methylation assay. After approximately 15 years of follow-up, 486 patients had died, and 186 of the deaths were breast cancer-related. We used Cox proportional hazards regression to estimate HRs and 95% CIs as well as likelihood ratio tests to assess multiplicative interactions.

**Results:**

All-cause mortality was lower only among physically active women with methylated promoter of *APC* (HR 0.60, 95% CI 0.40–0.80), *CCND2* (HR 0.56, 95% CI 0.32–0.99), *HIN* (HR 0.55, 95% CI 0.38–0.80), and *TWIST1* (HR 0.28, 95% CI 0.14–0.56) in tumors, but not among those with unmethylated tumors (significant interaction *p* < 0.05). We found no interaction between RPA and global methylation.

**Conclusions:**

The improved survival after breast cancer that is associated with RPA may be more pronounced in women with promoter tumor methylation in biologically plausible genes.

**Electronic supplementary material:**

The online version of this article (doi:10.1186/s13058-017-0811-z) contains supplementary material, which is available to authorized users.

## Background

With an estimated 40,000 deaths in 2017, breast cancer is the second leading cause of cancer-related death in the United States [1]. Women who engage in physical activity prior to the diagnosis of breast cancer have better overall survival than those who do not [2], but the mechanisms of this association are unknown. Given that only 20% of the U.S. population achieves the Centers for Disease Control and Prevention’s physical activity guidelines [3], improved understanding of how physical activity influences breast cancer prognosis could have significant public health impact.

Epigenetics is the study of functionally relevant changes to the genome that do not involve a change in the nucleotide sequence. DNA methylation is the most extensively studied epigenetic modification and involves the addition or removal of methyl (-CH_3_) groups at CpG dinucleotides that influence gene regulation [4]. DNA methylation can be measured in a range of tissues, including tumor and blood [5], and has been associated with breast cancer prognosis in several studies, including our own [6, 7]. Although methylation signatures are largely established during embryogenesis [8], DNA methylation (and other features of the epigenome) may be modified throughout the life course as a result of both behavioral and environmental stimuli [9], including physical activity [10]. Interactions between the environment and DNA methylation may, therefore, inform prognostic outcomes among women diagnosed with breast cancer.

In a population-based sample of women diagnosed with first primary breast cancer, we aimed to understand whether the association between prediagnostic recreational physical activity (RPA) and all-cause or breast cancer-specific mortality was modified by gene promoter methylation (which regulates gene expression) in a panel of 13 breast cancer-related genes (*APC*, *BRCA1*, *CCND2*, *CDH1*, *DAPK1*, *ESR1*, *GSTP1*, *HIN1*, *CDKN2A*, *PGR*, *RAR*β, *RASSF1A*, and *TWIST1*) measured in tumor tissue. Similarly, we sought to determine whether the RPA-mortality association was modified by global DNA methylation (a marker of genome stability) using two methods to assess white blood cell methylation: long interspersed nucleotide element 1 (LINE-1), which approximates levels in repetitive elements [11], and the luminometric methylation assay (LUMA), which estimates methylation at CCGG sites [12]. We hypothesized that methylation of oncogenes (or lack of methylation in tumor suppressor genes) and high prediagnostic physical activity engagement would result in lower all-cause and breast cancer-specific mortality among women diagnosed with first primary breast cancer. We also hypothesized that physical activity and low LUMA (high LINE-1) would work in synergy to reduce mortality following a breast cancer diagnosis.

## Methods

For this project, we used resources from the follow-up component of the Long Island Breast Cancer Study Project (LIBCSP), a population-based study. Details of the study design and participants for this component have been described previously [13, 14].

### Study participants

Eligible participants in the LIBCSP follow-up study were English-speaking female residents of Nassau and Suffolk counties on Long Island, NY, USA, who were newly diagnosed with a first primary in situ or invasive breast cancer between 1 August 1996 and 31 July 1997. Potentially eligible subjects were identified through daily or weekly contact with pathology departments of all 28 hospitals on Long Island and 3 tertiary care hospitals in New York City. At diagnosis, the 1508 women with breast cancer were aged 20–98 years, predominately postmenopausal (67%) and white (94%), which is consistent with the underlying racial/ethnic distribution in these two New York counties at the time of data collection.

### Data collection

#### Recreational physical activity and other covariates

Approximately 2–3 months after diagnosis, women were interviewed at home by trained interviewers using structured questionnaires. As part of this baseline (on average 100-minute) interview, RPA was assessed using a modified instrument developed by Bernstein and colleagues for epidemiologic studies of breast cancer [15]. RPA from menarche to diagnosis was used to estimate lifetime RPA, and women were classified as inactive, low RPA (<6.36 h/week), and high RPA (≥6.36 h/week) on the basis of the median for the entire cohort as previously described [16]. During the baseline interview, participants were additionally queried on their demographic characteristics (including age, race/ethnicity, income, and education), lifestyle characteristics (including cigarette smoking and body size), medical histories (including family history of breast cancer, exogenous hormone use, and mammography screening), and other breast cancer-related factors as previously described [13, 14].

#### Medical records data

Medical records were abstracted at baseline and again approximately 5 years later to determine tumor characteristics (e.g., estrogen receptor [ER]/progesterone receptor [PR] status, tumor size, and nodal involvement) as well as the first course of treatment for the first primary breast cancer diagnosis.

#### Gene-specific promoter methylation

DNA extraction from archived formalin-fixed, paraffin-embedded tumor tissue of the first primary breast cancer was performed as previously described [17]. Among the 975 women with archived tumor tissue, 807 (82.8%) had available gene promoter methylation data. The 807 women with tumor methylation data did not differ from the 1508 eligible women on most demographic and clinical characteristics. Women with tumor methylation data were more likely to have nodal involvement and invasive cancer (data not shown), which reflects the amount of tumor material available for methylation analyses.

Thirteen genes known to be involved in breast carcinogenesis, and frequently methylated in promoter regions, were selected for assessing interactions with RPA. Promoter methylation of *ESR1*, *PR*, and *BRCA1* was determined by methylation-specific (MSP) polymerase chain reaction (PCR) and was dichotomized (i.e., methylated vs. unmethylated) on the basis of the presence or absence of the PCR band [17, 18]. The methylation status of the ten remaining genes was assessed by the MethyLight assay (Qiagen, Valencia, CA, USA) [19, 20]. The percentage of methylation was calculated by the comparative cycle threshold (2^−ΔΔCT^) method, where ΔΔC_T_ = (C_T,Target_ − C_T,Actin_)_sample_ − (C_T,Target_ − C_T,Actin_)_fully methylated DNA_ [21], and multiplying by 100. Using a 4% cutoff, we dichotomized into methylated or unmethylated cases as previously reported [22].

#### Global methylation

For 1102 (73.1%) of women with breast cancer, trained phlebotomists obtained a nonfasting 40-ml blood sample at the baseline interview, and DNA was isolated as previously described [23]. Details of LUMA and LINE-1 assessment in the LIBCSP have been detailed previously [12]. Briefly, LUMA was carried out according to the modified protocol described by Bjornsson et al. [24] and was expressed as a percentage based on the following equation: methylation (%) = [1 − (HpaII ΣG/ΣT)/(MspI ΣG/ΣT)] × 100 [24]. Four CpG sites in the promoter region of LINE-1 were assessed using a prevalidated pyrosequencing-based methylation assay [19] and were individually analyzed as a T/C single-nucleotide polymorphism using Q-CpG software (Qiagen). These data were subsequently averaged to provide an overall percentage 5-methylcytosine status.

#### Mortality

We used the National Death Index to determine vital status through the end of 2011 as previously reported [25]. After approximately 14.7 (0.2–15.4) years of follow-up, among the 1254 patients with any gene-specific (range *n* = 726–803 women with gene promoter methylation status) or global methylation (range *n* = 1005–1015 women with LUMA or LINE-1) assessments and complete RPA data, we identified 421 who died as a result of any cause, of which 186 deaths were breast cancer-related (determined using International Classification of Diseases code 174.9 or C-50.9).

### Statistical analysis

We used Cox proportional hazards regression [26] to estimate HRs and 95% CIs for the association between RPA, methylation status (global and gene-specific), and mortality (all-cause and breast cancer-specific) among 1254 women with any methylation biomarker and complete RPA assessment. The 1254 women with breast cancer did not meaningfully differ from the original 1508 who were eligible. The women were more likely to have nodal involvement and invasive cancer, which relate to the amount of tumor material that would be available for assay. All statistical tests were two-sided (a priori significance level of 0.05). The proportional hazards assumption was assessed using exposure interactions with log-time [26]. We observed no violations of the proportional hazards assumption with the 13 breast-cancer related genes, global methylation markers, or RPA.

For interaction analyses, we assessed RPA using a three-level classification based on the median level among active participants: inactive, low RPA (<6.36 h/week), and high RPA (≥6.36 h/week). As detailed above, methylation of gene promoters was classified as methylated or unmethylated using a 4% cutoff, and global methylation markers (LUMA and LINE-1) were dichotomized at the median. Effect measure modification on the multiplicative scale between RPA and methylation was evaluated using the likelihood ratio test with a 0.05 significance level [27].

All models were initially adjusted for age at diagnosis. We further considered inclusion of family history of breast cancer (yes/no), history of benign breast disease (yes/no), cigarette smoking (ever/never), race (white, black, other), and body mass index (BMI; <25.0 kg/m^2^, 25.0–29.9 kg/m^2^, ≥30 kg/m^2^). Covariates were removed from the multivariate model using backward elimination. Variables remained in the final model if their exclusion changed the effect estimate by >10% [28]. None of these covariates met our criteria, and thus all models were adjusted for age at diagnosis only.

When constructing our models, we did not consider tumor characteristics (e.g., tumor stage, grade, size, and nodal involvement) or hormone receptor status as potential confounders of the association between RPA, methylation, and mortality. These covariates are on the causal pathway between prediagnostic RPA and mortality, and adjustment for a causal intermediate would result in biased parametric estimates [29, 30]. Although our study population includes women with invasive (84%) and in situ (16%) breast cancer, our findings restricted to invasive tumors did not vary substantially from those among all women, likely owing to the lower proportion of in situ cases in our study population. We therefore considered both invasive and noninvasive cases in these analyses. All statistical analyses were performed using SAS statistical software version 9.4 (SAS Institute, Cary, NC, USA).

## Results

### Distribution of clinical characteristics

The distribution of clinical characteristics by RPA category among the 1254 women with breast cancer included in this study are provided in Table 1. The distribution of clinical characteristics by outcome (all-cause and breast cancer-specific mortality) is available in Additional file 1: Table S1. Women who engaged in RPA across the life course tended to have younger age at diagnosis and a lower BMI, and they were slightly less likely to have nodal involvement. We found little difference in other clinical characteristics (i.e., ER or PR status) among physically active women compared with inactive women.

**Table 1:** Distribution of clinical characteristics by recreational physical activity category among the 1254 participants with any information on methylation (gene-specific and/or global) and lifetime physical activity in a population-based cohort of women diagnosed with first primary breast cancer, Long Island Breast Cancer Study Project

Recreational physical activity	Inactive (*n* = 294)	<6.36 h/week (*n* = 497)	≥6.36 h/week (*n* = 463)
	*n* (%)	*n* (%)	*n* (%)
Age at diagnosis			
<50 years	55 (18.71)	170 (34.21)	136 (29.37)
≥50 years	239 (81.29)	327 (65.79)	327 (70.63)
Menopausal status			
Premenopausal	55 (19.37)	187 (38.09)	148 (32.74)
Postmenopausal	229 (80.63)	304 (61.91)	304 (67.26)
Family history of breast cancer			
No	225 (78.13)	396 (82.16)	361 (80.94)
Yes	63 (21.88)	86 (17.84)	85 (19.06)
Body mass index (BMI)			
BMI <25 kg/m^2^	113 (38.97)	240 (48.58)	200 (43.67)
BMI 25–29.9 kg/m^2^	92 (31.72)	157 (31.78)	157 (34.28)
BMI ≥30 kg/m^2^	85 (29.31)	97 (19.64)	191 (22.05)
Cigarette smoking			
Never	138 (46.94)	231 (46.48)	199 (42.98)
Current/former	156 (53.06)	266 (53.32)	264 (57.02)
History of benign breast disease			
No	248 (84.35)	393 (79.07)	370 (80.09)
Yes	46 (15.65)	104 (20.93)	92 (19.91)
Cancer type			
In situ	39 (13.27)	97 (19.52)	57 (12.31)
Invasive	255 (86.73)	400 (80.48)	406 (87.69)
Hormone receptor status^a^			
Positive	158 (81.44)	252 (78.02)	260 (79.75)
Negative	36 (18.35)	71 (21.98)	66 (20.25)
Estrogen receptor status			
Positive	147 (75.77)	235 (72.76)	248 (76.07)
Negative	47 (24.23)	88 (27.24)	78 (23.93)
Progesterone receptor status			
Positive	125 (64.43)	203 (62.85)	220 (67.48)
Negative	69 (35.57)	120 (37.15)	106 (32.52)
Tumor size			
<2 cm	94 (60.26)	171 (66.28)	187 (68.75)
≥2 cm	62 (39.74)	87 (33.72)	85 (31.25)
Nodal involvement			
0	45 (27.78)	73 (27.76)	48 (18.05)
1	117 (72.22)	190 (72.24)	218 (81.95)
Treatment type			
No chemotherapy	120 (65.57)	206 (56.59)	183 (60.00)
Chemotherapy	63 (34.43)	158 (43.41)	122 (40.00)
No radiation	75 (40.98)	148 (40.55)	113 (36.81)
Radiation	108 (59.02)	217 (59.45)	194 (63.19)
No hormone therapy	58 (32.22)	144 (40.11)	119 (39.40)
Hormone therapy	122 (67.78)	215 (59.89)	183 (60.60)

^a^ Any estrogen receptor-positive or progesterone receptor-positive tumor

### Associations between RPA and all-cause and breast cancer-specific mortality

In Table 2, we provide effect estimates for the association between prediagnostic lifetime RPA and mortality after approximately 15 years of follow-up among our LIBCSP cohort of 1254 women newly diagnosed with first primary breast cancer in 1996–1997. The association between lifetime RPA and mortality among the entire cohort of 1508 women with breast cancer with follow-up through 2002 was previously reported [14]; follow-up has now been updated and extended through 2011. Our updated estimates showing inverse associations with both all-cause and breast cancer-specific morality are similar to the earlier reported estimates in the LIBCSP based on 5 years of follow-up. The biological relevance and function of the 13 genes investigated in this study [31] are summarized in Additional file 2: Table S2, along with previously reported associations with RPA [32] and breast cancer-specific mortality [33].

**Table 2:** Age-adjusted HRs and 95% CIs for the association between lifetime recreational physical activity and 15-year all-cause and breast cancer-specific mortality among a population-based sample of 1254 women with a first primary breast cancer, Long Island Breast Cancer Study Project

	All-cause mortality			Breast cancer-specific mortality		
Recreational physical activity^a^						
Inactive	129/294	1.00	Reference	55/294	1.00	Reference
<6.36 h/week	144/497	0.71	(0.56–0.90)	66/497	0.60	(0.42–0.86)
≥ 6.36 h/week	148/463	0.75	(0.59–0.94)	65/463	0.66	(0.46–0.95)

^a^Cleveland et al. [14] previously reported age-, BMI-, and menopausal status-adjusted associations for prediagnostic recreational physical activity (measured as metabolic equivalents of task-h/week), with follow-up though 2002

### Associations between RPA, DNA methylation, and mortality

**Fig. 1:**
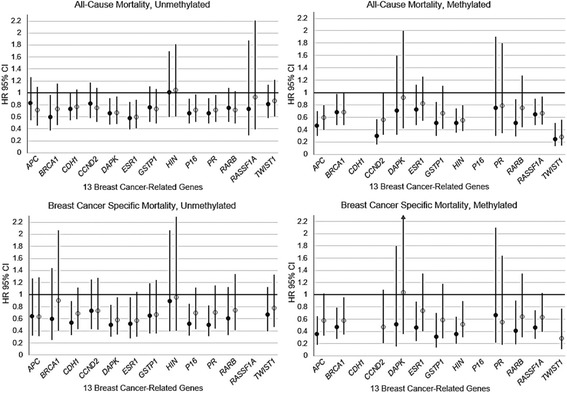
Age-adjusted HRs and 95% CIs for the association between lifetime recreational physical activity (RPA) and 15-year all-cause and breast cancer-specific mortality, stratified by gene methylation status (methylated vs. unmethylated tumors), among 803 women diagnosed with a first primary breast cancer and with available gene promoter methylation data, Long Island Breast Cancer Study Project. *Closed circles* = low RPA (<6.36 h/week). *Open circles* = high RPA (≥6.36 h/week). Compared with inactive women (data point not shown, HR 1.0)

As shown in Table 3, the association between prediagnostic lifetime RPA and all-cause mortality following a breast cancer diagnosis was lower among active women (>6.36 h/week of RPA) with breast tumor promoter methylation in *APC* (HR 0.60, 95% CI 0.40–0.80), *CCND2* (HR 0.56, 95% CI 0.32–0.99), *HIN* (HR 0.55, 95% CI 0.38–0.80), and *TWIST1* (HR 0.28, 95% CI 0.14–0.56), but not among active women with unmethylated tumors (*p* < 0.05 for multiplicative interaction). Overall, we found substantially lower risk of all-cause mortality among women with any RPA and methylated gene promoters than among active women with unmethylated promoters (Fig. 1). For example, we observed an almost 50% lower risk of death as a result of all causes among very active women with methylated *HIN1* promoter (HR 0.55, 95% CI 0.38–0.80). In contrast, there was no corresponding risk reduction for RPA among those with unmethylated *HIN1* promoter (HR 1.09, 95% CI 0.61–1.81). We observed similar patterns of association for breast cancer-specific mortality, albeit the interaction was not significant (RPA HR 0.96, 95% CI 0.40–2.29 for unmethylated *HIN1* vs. RPA HR 0.52, 95% CI 0.30–0.90 for unmethylated *HIN1*; multiplicative interaction *p* = 0.066). We did not observe an interaction between RPA, *APC* methylation, and breast cancer-specific mortality (*p* = 0.138). For *CCND2* and *TWIST1*, we were unable to evaluate effect modification owing to small cells.

**Table 3:** Age-adjusted HRs and 95% CIs for the association between lifetime recreational physical activity and 15-year all-cause and breast cancer-specific mortality stratified by gene methylation status (methylated vs. unmethylated tumors) among 803 women diagnosed with a first primary breast cancer and with available gene promoter methylation data, Long Island Breast Cancer Study Project

	All-cause mortality						Breast cancer-specific mortality					
	Unmethylated			Methylated			Unmethylated			Methylated		
Gene promoter and RPA categories	Deaths/cases–*n*	HR	95% CI	Deaths/cases–*n*	HR	95% CI	Deaths/cases–*n*	HR	95% CI	Deaths/cases–*n*	HR	95% CI
*APC*												
Inactive	38/88	1.00	Reference	49/93	1.00	Reference	14/88	1.00	Reference	24/93	1.00	Reference
<6.36 h/week	54/173	0.83	(0.54–1.26)	38/135	0.46	(0.30–0.70)	21/173	0.64	(0.32–1.27)	16/135	0.35	(0.18–0.65)
≥6.36 h/week	40/131	0.71	(0.45–1.10)	46/136	0.60	(0.40–0.80)	16/131	0.63	(0.31–1.29)	26/136	0.58	(0.33–1.02)
*p*-interaction	0.038						0.138					
*BRCA1*												
Inactive	35/79	1.00	Reference	56/118	1.00	Reference	9/79	1.00	Reference	30/118	1.00	Reference
<6.36 h/week	33/128	0.60	(0.37–0.97)	63/192	0.68	(0.47–0.98)	12/128	0.60	(0.25–1.44)	27/192	0.47	(0.28–0.79)
≥ 6.36 h/week	38/119	0.73	(0.46–1.16)	56/167	0.68	(0.47–0.99)	15/119	0.90	(0.40–2.07)	29/167	0.58	(0.35–0.96)
*p*-interaction	0.485						0.341					
*CDH1*												
Inactive	69/157	1.00	Reference	11/16	1.00	Reference	30/157	1.00	Reference	<5/16	Not estimated	
< 6.36 h/week	88/283	0.73	(0.54–1.01)	<5/15	Not estimated^a^		35/283	0.54	(0.33–0.89)	<5/15	Not estimated	
≥6.36 h/week	81/243	0.77	(0.56–1.06)	<5/12	Not estimated		37/243	0.69	(0.43–1.12)	<5/12	Not estimated	
*p*-interaction	Not estimated						Not estimated					
*CCND2*												
Inactive	55/135	1.00	Reference	25/38	1.00	Reference	22/135	1.00	Reference	12/38	1.00	Reference
< 6.36 h/week	76/244	0.82	(0.58–1.17)	15/54	0.30	(0.16–0.57)	34/244	0.73	(0.43–1.25)	<5/54	not estimated	
≥6.36 h/week	61/202	0.75	(0.52–1.08)	24/53	0.56	(0.32–0.99)	28/202	0.73	(0.42–1.28)	11/53	0.47	(0.21–1.08)
*p*-interaction	0.004						Not estimated					
*DAPK*												
Inactive	69/149	1.00	Reference	11/24	1.00	Reference	29/149	1.00	Reference	5/24	1.00	Reference
< 6.36 h/week	78/259	0.66	(0.47–0.91)	13/39	0.71	(0.32–1.60)	31/259	0.50	(0.30–0.83)	5/39	0.52	(0.15–1.80)
≥6.36 h/week	70/216	0.67	(0.48–0.94)	15/39	0.92	(0.42–2.00)	29/216	0.58	(0.34–0.96)	10/39	1.04	(0.36–3.05)
*p*-interaction	0.467						0.247					
*ESR1*												
Inactive	51/104	1.00	Reference	40/91	1.00	Reference	19/104	1.00	Reference	20/91	1.00	Reference
< 6.36 h/week	53/178	0.58	(0.39–0.85)	42/138	0.73	(0.47–1.13)	23/178	0.52	(0.28–0.96)	16/138	0.46	(0.24–0.89)
≥6.36 h/week	49/155	0.60	(0.41–0.89)	45/129	0.82	(0.54–1.26)	21/155	0.57	(0.30–1.05)	23/129	0.74	(0.40–1.35)
*p*-interaction	0.298						0.353					
*GSTP1*												
Inactive	47/121	1.00	Reference	33/52	1.00	Reference	19/121	1.00	Reference	15/52	1.00	Reference
< 6.36 h/week	64/220	0.76	(0.52–1.11)	27/78	0.51	(0.30–0.85)	26/220	0.65	(0.36–1.19)	10/78	0.31	(0.14–0.70)
≥6.36 h/week	54/182	0.73	(0.49–1.07)	31/73	0.67	(0.41–1.11)	22/182	0.67	(0.36–1.24)	17/73	0.59	(0.29–1.18)
*p*-interaction	0.219						0.134					
*HIN*												
Inactive	24/67	1.00	Reference	56/106	1.00	Reference	9/67	1.00	Reference	25/106	1.00	Reference
< 6.36 h/week	36/113	1.01	(0.60–1.70)	55/185	0.51	(0.35–0.74)	15/113	0.89	(0.39–2.07)	21/185	0.36	(0.20–0.64)
≥6.36 h/week	29/88	1.05	(0.61–1.81)	56/167	0.55	(0.38–0.80)	12/88	0.96	(0.40–2.29)	27/167	0.52	(0.30–0.90)
*p*-interaction	0.016						0.066					
*P16*												
Inactive	76/166	1.00	Reference	9/11	1.00	Reference	31/166	1.00	Reference	7/11	1.00	Reference
< 6.36 h/week	88/293	0.66	(0.49–0.90)	<5/9	Not estimated		35/293	0.52	(0.32–0.85)	<5/9	Not estimated	
≥6.36 h/week	82/245	0.71	(0.52–0.98)	<5/10	Not estimated		38/245	0.70	(0.43–1.12)	<5/10	Not estimated	
*p*-interaction	Not estimated						Not estimated					
*PR*												
Inactive	76/174	1.00	Reference	15/23	1.00	Reference	31/174	1.00	Reference	8/23	1.00	Reference
< 6.36 h/week	87/292	0.66	(0.49–0.91)	9/28	0.75	(0.30–1.90)	32/292	0.50	(0.31–0.82)	7/28	0.67	(0.22–2.10)
≥6.36 h/week	80/245	0.71	(0.52–0.97)	14/41	0.79	(0.34–1.80)	36/245	0.71	(0.44–1.15)	8/41	0.55	(0.18–1.64)
*p*-interaction	0.893						0.226					
*RARB*												
Inactive	52/114	1.00	Reference	28/59	1.00	Reference	18/114	1.00	Reference	16/59	1.00	Reference
< 6.36 h/week	70/225	0.75	(0.52–1.08)	21/73	0.51	(0.29–0.89)	26/225	0.61	(0.33–1.13)	10/73	0.41	(0.19–0.91)
≥6.36 h/week	59/189	0.71	(0.49–1.03)	26/66	0.75	(0.44–1.28)	27/189	0.74	(0.41–1.34)	12/66	0.64	(0.30–1.35)
*p*-interaction	0.158						0.420					
*RASSF1A*												
Inactive	8/25	1.00	Reference	72/148	1.00	Reference	<5/25	Not estimated		31/148	1.00	Reference
< 6.36 h/week	10/38	0.73	(0.29–1.88)	81/260	0.65	(0.47–0.90)	5/38	Not estimated		31/260	0.46	(0.28–0.75)
≥6.36 h/week	15/47	0.93	(0.39–2.21)	70/208	0.67	(0.48–0.94)	5/47	Not estimated		34/208	0.63	(0.39–1.03)
*p*-interaction	0.330						Not estimated					
*TWIST1*												
Inactive	60/148	1.00	Reference	20/25	1.00	Reference	25/148	1.00	Reference	9/25	1.00	Reference
< 6.36 h/week	78/257	0.81	(0.58–1.14)	13/41	0.25	(0.13–0.51)	32/257	0.67	(0.39–1.13)	<5/41	Not estimated	
≥6.36 h/week	72/214	0.87	(0.61–1.22)	13/41	0.28	(0.14–0.56)	30/214	0.78	(0.46–1.33)	9/41	0.29	(0.11–0.77)
*p*-interaction	0.001						Not estimated					

^a^ Point estimate was not calculated, because cell sizes were less than 5

When we restricted our analyses to women with hormone receptor-positive breast cancer only (defined as any ER- or PR-positive), our estimates became less precise but were similar in effect size, and most interactions persisted (Additional file 3: Table S3). The association between RPA and mortality among women with breast cancer was not modified by global methylation markers, LINE-1, or LUMA (Additional file 4: Table S4).

## Discussion

In this population-based follow-up study of 1254 women diagnosed with first primary breast cancer, we found that the overall improved survival among women with any lifetime prediagnostic RPA appeared to be modified by gene-specific methylation profiles. We observed substantially improved survival with high lifetime prediagnostic RPA in women with a tumor-methylated *APC*, *CCND2*, *HIN1*, or *TWIST1* gene promoter compared with active women with unmethylated gene promoters. A more pronounced risk reduction was observed for breast cancer-specific mortality for the interaction with *HIN1*; however, we were unable to evaluate interactions with breast cancer-specific mortality owing to small numbers. We found no interaction between RPA and global methylation as measured by LINE-1 and LUMA. Our findings suggest that the inverse association between RPA and mortality after breast cancer may depend upon gene-specific methylation profiles.

Improved survival with RPA among women with breast cancer has been observed in many epidemiologic studies [2], including our own [14]. Also, we [6] and others have reported associations between gene-specific methylation and prognosis [34]. However, to our knowledge, no previous investigation has considered gene methylation as a potential modifier of the RPA-mortality association, despite strong biologic plausibility. Not only does physical activity reduce adiposity and its numerous metabolic correlates, but it is itself thought to reduce markers of inflammation, alter immune functioning, and lower circulating insulin [35, 36]. These pathways have been linked to aberrant DNA methylation, altering several genes implicated in breast carcinogenesis [37–40]. Collectively, these data suggest that the mechanisms underlying the inverse association between RPA and mortality may be facilitated and/or altered by inflammation-related methylation changes.

In the present study, we found that the improved survival after breast cancer with high RPA was greatest among patients with methylated *APC*, *CCND2*, *HIN1*, and *TWIST1* promoters. *APC* and *HIN1* are candidate tumor suppressors thought to be involved in breast carcinogenesis [41, 42]. Our observation of decreased mortality among very active women with *APC* or *HIN1* methylation is counter to our a priori hypothesis of lowered risk of death among active women with unmethylated (active) tumor suppressor genes, although we did observe that in the highest RPA group, there was no statistical difference in the effect among *APC* methylated and unmethylated cases. We observed risk reductions at both low and high RPA engagement among women with methylated *HIN1* promoters. Methylation of *HIN1* is linked to gene silencing, reduced expression, and loss of apoptosis [43]. *HIN1* is a putative growth inhibitory cytokine thought to be inactivated at the earliest stages of breast tumorigenesis and silenced in the majority of sporadic breast carcinomas [44]. This may suggest that prediagnostic RPA could help overcome the deleterious effects of *HIN1* inactivation in breast carcinogenesis, thereby improving survival outcomes.

The exact roles of *CCND2* and *TWIST1* in breast cancer are unresolved. *CCND2* is important in cell cycle regulation and has been cited as both a tumor suppressor gene [45] and an oncogene [46]. Inactivation of *CCND2* is thought to occur via promoter hypermethylation, which may be an early, though infrequent (about 11%), event in malignant breast cancer transformation [45, 47, 48]. In our study, we found lower all-cause mortality among women with promoter methylation (or loss of *CCND2* expression) in tumor tissue. This may reflect synergy between physical exercise and inactivation of the *CCND2* oncogene, particularly among women with low RPA. *TWIST1* is an antiapoptotic and prometastatic transcription factor, and methylation of the gene promoter has been observed frequently in malignant breast tissue [49]. We observed pronounced reductions in all-cause mortality among physically active patients with *TWIST1* methylation, which is consistent with our a priori hypothesis of synergy between the presumptive oncogene and RPA.

Although our population-based study of women with breast cancer was carefully conducted and included comprehensive exposure assessment and a long follow-up time, several potential limitations should be considered. First, information on RPA was collected systematically by trained interviewers [13]; nonetheless, there is potential for nondifferential measurement error, which would result in reduced effect estimates. However, LIBCSP investigators used a comprehensive, open-ended approach to query women on their lifetime RPA habits. This approach has been shown to elucidate important relationships between RPA and breast cancer in the LIBCSP [14, 16] and is consistent with other findings [50]. Second, postdiagnostic RPA, which likely influences prognosis [51], was not considered in this investigation, owing to small cells after stratification by methylation status. Third, we were limited to a panel of 13 biologically relevant genes and 2 global assays. However, studies employing more robust panels that interrogate hundreds of thousands of CpG sites are at high risk for false-positives, generally lack biologically driven hypotheses, and perform modestly using archived tumor samples [52, 53]. Additionally, we were limited by the use of conventional MSP PCR assays for three of the genes. However, where quantitative MSP PCR assays have the advantage of providing a quantitative estimate of methylation, the conventional MSP assay is a highly sensitive method to classify individuals by methylation status, which mitigates the threat of biased results [54, 55]. Finally, the racial homogeneity of our study population restricted our ability to explore potential variation by intrinsic subtype or by race, both of which are known to associate with prognostic outcomes [56]. Nonetheless, the largest hormonal subtype of breast cancer diagnosed among U.S. women of any race is ER^+^PR^+^ [57], which continues to increase with time [58] and is the predominant subtype of breast cancer diagnosed among LIBCSP study participants. When we restricted our findings to women with only hormone-responsive breast tumors, results were similar to those for all women.

## Conclusions

To our knowledge, we are the first to show, using resources from a population-based follow-up study, that promoter methylation of *APC*, *CCND2*, *HIN1*, and *TWIST1* may modify the inverse association between prediagnostic RPA and all-cause mortality following a breast cancer diagnosis. With the exception of *HIN1*, which was suggestive of breast cancer-specific mortality, power was limited for examining potential modification of the association between RPA and breast cancer-specific mortality. Although our results require confirmation in cohort studies with a larger number of women with breast cancer and more comprehensive gene coverage, they suggest that DNA methylation may play an important role in associations between physical activity and improved survival among women with breast cancer.

## Additional files

Additional file 1: Table S1. Distribution of clinical characteristics by mortality status among the 1254 participants with any information on methylation (gene-specific and/or global) and lifetime physical activity in a population-based cohort of women diagnosed with first primary breast cancer, Long Island Breast Cancer Study Project. (DOC 59 kb)
Additional file 2: Table S2. Gene function and previously observed methylation status of the 13 investigated genes in relation to recreational physical activity (RPA) and breast cancer-specific mortality, Long Island Breast Cancer Study Project. (DOC 36 kb)
Additional file 3: Table S3. Age-adjusted HRs and 95% CIs for the association between lifetime recreational physical activity (RPA) and 15-year all-cause and breast cancer-specific mortality stratified by gene methylation status (methylated vs. unmethylated tumors) among 803 women diagnosed with a first primary hormone receptor-positive breast cancer and with available gene promoter methylation data, Long Island Breast Cancer Study Project. (DOC 56 kb)
Additional file 4: Table S4. Age-adjusted HRs and 95% CIs for the association between lifetime recreational physical activity (RPA) and 15-year all-cause and breast cancer-specific mortality, stratified by global methylation status (measured by LUMA and LINE-1), among a population-based sample of 1015 women diagnosed with a first primary breast cancer and with available global methylation data, Long Island Breast Cancer Study Project. (DOC 43 kb)

## Abbreviations

2^−ΔΔCT^: Comparative cycle threshold method
BMI: Body mass index
ER: Estrogen receptor
LIBCSP: Long Island Breast Cancer Study Project
LINE-1: Long interspersed nucleotide element 1
LUMA: Luminometric methylation assay
MSP: Methylation-specific
PCR: Polymerase chain reaction
PR: Progesterone receptor
RPA: Recreational physical activity

## References

[1] American Cancer Society. Breast cancer. http://www.cancer.org/cancer/breastcancer/detailedguide/breast-cancer-key-statistics. Accessed 28 June 2016.

[2] Friedenreich CM (2010). The role of physical activity in breast cancer etiology. Semin Oncol.

[3] Centers for Disease Control and Prevention (CDC). Facts about physical activity. http://www.cdc.gov/physicalactivity/data/facts.htm. Accessed 30 June 2016.

[4] Fan S, Zhang X (2009). CpG island methylation pattern in different human tissues and its correlation with gene expression. Biochem Biophys Res Commun.

[5] van Hoesel AQ, van de Velde CJ, Kuppen PJ, Liefers GJ, Putter H, Sato Y (2012). Hypomethylation of LINE-1 in primary tumor has poor prognosis in young breast cancer patients: a retrospective cohort study. Breast Cancer Res Treat.

[6] Xu X, Gammon MD, Zhang Y, Cho YH, Wetmur JG, Bradshaw PT (2010). Gene promoter methylation is associated with increased mortality among women with breast cancer. Breast Cancer Res Treat.

[7] Cho YH, Shen J, Gammon MD, Zhang YJ, Wang Q, Gonzalez K (2012). Prognostic significance of gene-specific promoter hypermethylation in breast cancer patients. Breast Cancer Res Treat.

[8] Nagase H, Ghosh S (2008). Epigenetics: differential DNA methylation in mammalian somatic tissues. FEBS J.

[9] Feil R, Fraga MF (2012). Epigenetics and the environment: emerging patterns and implications. Nat Rev Genet.

[10] White AJ, Sandler DP, Bolick SC, Xu Z, Taylor JA, DeRoo LA (2013). Recreational and household physical activity at different time points and DNA global methylation. Eur J Cancer.

[11] Weisenberger DJ, Campan M, Long TI, Kim M, Woods C, Fiala E (2005). Analysis of repetitive element DNA methylation by MethyLight. Nucleic Acids Res.

[12] Xu X, Gammon MD, Hernandez-Vargas H, Herceg Z, Wetmur JG, Teitelbaum SL (2012). DNA methylation in peripheral blood measured by LUMA is associated with breast cancer in a population-based study. FASEB J.

[13] Gammon MD, Neugut AI, Santella RM, Teitelbaum SL, Britton JA, Terry MB (2002). The Long Island Breast Cancer Study Project: description of a multi-institutional collaboration to identify environmental risk factors for breast cancer. Breast Cancer Res Treat.

[14] Cleveland RJ, Eng SM, Stevens J, Bradshaw PT, Teitelbaum SL, Neugut AI (2012). Influence of prediagnostic recreational physical activity on survival from breast cancer. Eur J Cancer Prev.

[15] Bernstein M, Sloutskis D, Kumanyika S, Sparti A, Schutz Y, Morabia A (1998). Data-based approach for developing a physical activity frequency questionnaire. Am J Epidemiol.

[16] McCullough LE, Eng SM, Bradshaw PT, Cleveland RJ, Teitelbaum SL, Neugut AI (2012). Fat or fit: the joint effects of physical activity, weight gain, and body size on breast cancer risk. Cancer.

[17] Xu X, Gammon MD, Zhang Y, Bestor TH, Zeisel SH, Wetmur JG (2009). BRCA1 promoter methylation is associated with increased mortality among women with breast cancer. Breast Cancer Res Treat.

[18] Liu ZJ, Maekawa M, Horii T, Morita M (2003). The multiple promoter methylation profile of PR gene and ERα gene in tumor cell lines. Life Sci.

[19] Eads CA, Danenberg KD, Kawakami K, Saltz LB, Danenberg PV, Laird PW (1999). CpG island hypermethylation in human colorectal tumors is not associated with DNA methyltransferase overexpression. Cancer Res.

[20] Eads CA, Lord RV, Kurumboor SK, Wickramasinghe K, Skinner ML, Long TI (2000). Fields of aberrant CpG island hypermethylation in Barrett’s esophagus and associated adenocarcinoma. Cancer Res.

[21] Livak KJ, Schmittgen TD (2001). Analysis of relative gene expression data using real-time quantitative PCR and the 2^−ΔΔ*C*^_T_ method. Methods.

[22] Eads CA, Danenberg KD, Kawakami K, Saltz LB, Blake C, Shibata D (2000). MethyLight: a high-throughput assay to measure DNA methylation. Nucleic Acids Res.

[23] Gammon MD, Sagiv SK, Eng SM, Shantakumar S, Gaudet MM, Teitelbaum SL (2004). Polycyclic aromatic hydrocarbon-DNA adducts and breast cancer: a pooled analysis. Arch Environ Health.

[24] Bjornsson HT, Sigurdsson MI, Fallin MD, Irizarry RA, Aspelund T, Cui H (2008). Intra-individual change over time in DNA methylation with familial clustering. JAMA.

[25] Khankari NK, Bradshaw PT, Steck SE, He K, Olshan AF, Shen J (2015). Dietary intake of fish, polyunsaturated fatty acids, and survival after breast cancer: a population-based follow-up study on Long Island, New York. Cancer.

[26] Allison PD (2010). Survival analysis using SAS: a practical guide.

[27] Kleinbaum D, Klein M (2002). Logistic regression: a self-learning text.

[28] Hosmer DW, Lemeshow S (1989). Applied logistic regression. Wiley series in probability and mathematical statistics: applied probability and statistics. 2nd ed.

[29] Cole SR, Hernan MA (2002). Fallibility in estimating direct effects. Int J Epidemiol.

[30] Schisterman EF, Cole SR, Platt RW (2009). Overadjustment bias and unnecessary adjustment in epidemiologic studies. Epidemiology.

[31] Xu X, Gammon MD, Jefferson E, Zhang Y, Cho YH, Wetmur JG (2011). The influence of one-carbon metabolism on gene promoter methylation in a population-based breast cancer study. Epigenetics.

[32] McCullough LE, Chen J, White AJ, Xu X, Cho YH, Bradshaw PT, et al. Gene-specific promoter methylation status in hormone-receptor-positive breast cancer associates with postmenopausal body size and recreational physical activity. Int J Cancer Clin Res. 2015;2(1):013. 10.23937/2378-3419/2/1/1013 PMC4440485 26005715

[33] McCullough LE, Chen J, Cho YH, Khankari NK, Bradshaw PT, White AJ (2016). DNA methylation modifies the association between obesity and survival after breast cancer diagnosis. Breast Cancer Res Treat.

[34] Győrffy B, Bottai G, Fleischer T, Munkácsy G, Budczies J, Paladini L (2016). Aberrant DNA methylation impacts gene expression and prognosis in breast cancer subtypes. Int J Cancer.

[35] Mattusch F, Dufaux B, Heine O, Mertens I, Rost R (2000). Reduction of the plasma concentration of C-reactive protein following nine months of endurance training. Int J Sports Med.

[36] Hoffman-Goetz L, Apter D, Demark-Wahnefried W, Goran MI, McTiernan A, Reichman ME (1998). Possible mechanisms mediating an association between physical activity and breast cancer. Cancer.

[37] Starlard-Davenport A, Tryndyak VP, James SR, Karpf AR, Latendresse JR, Beland FA (2010). Mechanisms of epigenetic silencing of the *Rassf1a* gene during estrogen-induced breast carcinogenesis in ACI rats. Carcinogenesis.

[38] Fernandez SV, Snider KE, Wu YZ, Russo IH, Plass C, Russo J (2010). DNA methylation changes in a human cell model of breast cancer progression. Mutat Res.

[39] Kang GH, Lee HJ, Hwang KS, Lee S, Kim JH, Kim JS (2003). Aberrant CpG island hypermethylation of chronic gastritis, in relation to aging, gender, intestinal metaplasia, and chronic inflammation. Am J Pathol.

[40] Liggett T, Melnikov A, Yi QL, Replogle C, Brand R, Kaul K (2010). Differential methylation of cell-free circulating DNA among patients with pancreatic cancer versus chronic pancreatitis. Cancer.

[41] Jin Z, Tamura G, Tsuchiya T, Sakata K, Kashiwaba M, Osakabe M (2001). *Adenomatous polyposis coli* (*APC*) gene promoter hypermethylation in primary breast cancers. Br J Cancer.

[42] Krop I, Parker MT, Bloushtain-Qimron N, Porter D, Gelman R, Sasaki H (2005). HIN-1, an inhibitor of cell growth, invasion, and AKT activation. Cancer Res.

[43] Xu JH, Hu SL, Shen GD, Shen G. Tumor suppressor genes and their underlying interactions in paclitaxel resistance in cancer therapy. Cancer Cell Int. 2016;16:13. doi:10.1186/s12935-016-0290-9. 10.1186/s12935-016-0290-9 PMC4761208 26900348

[44] Krop I, Maguire P, Lahti-Domenici J, Lodeiro G, Richardson A, Johannsdottir HK (2003). Lack of HIN-1 methylation in BRCA1-linked and “BRCA1-like” breast tumors. Cancer Res.

[45] Virmani A, Rathi A, Heda S, Sugio K, Lewis C, Tonk V (2003). Aberrant methylation of the *cyclin D2* promoter in primary small cell, nonsmall cell lung and breast cancers. Int J Cancer.

[46] Zhang P (1999). The cell cycle and development: redundant roles of cell cycle regulators. Curr Opin Cell Biol.

[47] Evron E, Umbricht CB, Korz D, Raman V, Loeb DM, Niranjan B (2001). Loss of cyclin D2 expression in the majority of breast cancers is associated with promoter hypermethylation. Cancer Res.

[48] Li S, Rong M, Iacopetta B (2006). DNA hypermethylation in breast cancer and its association with clinicopathological features. Cancer Lett.

[49] Widschwendter M, Jones PA (2002). DNA methylation and breast carcinogenesis. Oncogene.

[50] Lahart IM, Metsios GS, Nevill AM, Carmichael AR (2015). Physical activity, risk of death and recurrence in breast cancer survivors: a systematic review and meta-analysis of epidemiological studies. Acta Oncol.

[51] Bradshaw PT, Ibrahim JG, Khankari N, Cleveland RJ, Abrahamson PE, Stevens J (2014). Post-diagnosis physical activity and survival after breast cancer diagnosis: the Long Island Breast Cancer Study. Breast Cancer Res Treat.

[52] Birney E, Smith GD, Greally JM (2016). Epigenome-wide association studies and the interpretation of disease -omics. PLoS Genet.

[53] Siegel EM, Berglund AE, Riggs BM, Eschrich SA, Putney RM, Ajidahun AO (2014). Expanding epigenomics to archived FFPE tissues: an evaluation of DNA repair methodologies. Cancer Epidemiol Biomarkers Prev.

[54] Hoque MO, Begum S, Topaloglu O, Jeronimo C, Mambo E, Westra WH (2004). Quantitative detection of promoter hypermethylation of multiple genes in the tumor, urine, and serum DNA of patients with renal cancer. Cancer Res.

[55] Lo YD, Wong IH, Zhang J, Tein MS, Ng MH, Hjelm NM (1999). Quantitative analysis of aberrant p16 methylation using real-time quantitative methylation-specific polymerase chain reaction. Cancer Res.

[56] O’Brien KM, Cole SR, Tse CK, Perou CM, Carey LA, Foulkes WD (2010). Intrinsic breast tumor subtypes, race, and long-term survival in the Carolina Breast Cancer Study. Clin Cancer Res.

[57] Clarke CA, Keegan TH, Yang J, Press DJ, Kurian AW, Patel AH (2012). Age-specific incidence of breast cancer subtypes: understanding the black-white crossover. J Natl Cancer Inst.

[58] Anderson WF, Katki HA, Rosenberg PS (2011). Incidence of breast cancer in the United States: current and future trends. J Natl Cancer Inst.

