# Supplementary material for: Modification of the association between recreational physical activity and survival after breast cancer by promoter methylation in breast cancer-related genes
Source: Breast Cancer Res. 2017 Feb 21;19:19. doi: 10.1186/s13058-017-0811-z (PMC5319077; doi:10.1186/s13058-017-0811-z)
Supplement: Additional file 1: Table S1. — Distribution of clinical characteristics by mortality status among the 1254 participants with any information on methylation (gene-specific and/or global) and lifetime physical activity in a population-based cohort of women diagnosed with first primary breast cancer, Long Island Breast Cancer Study Project. (DOC 59 kb) [file 13058_2017_811_MOESM1_ESM.doc]

| **Additional file 1: Table S1.** Distribution of clinical characteristics by mortality status among the 1254 participants with any information on methylation (gene-specific and/or global) and lifetime physical activity in a population-based cohort of women diagnosed with first primary breast cancer, Long Island Breast Cancer Study Project. | | | |
| --- | --- | --- | --- |
| **Mortality** | Alive  (N=833) | All-cause mortality (N=421) | Breast cancer-specific mortality (N=186) |
|  | N (%) | N (%) | N (%) |
| Age at diagnosis |  |  |  |
| <50 years | 283 (33.97) | 78 (18.53) | 59 (31.72) |
| ≥50 years | 550 (66.03) | 343 (81.47) | 127 (68.28) |
| Menopausal status |  |  |  |
| premenopausal | 306 (37.68) | 84 (20.24) | 60 (32.79) |
| postmenopausal | 506 (62.32) | 331 (79.76) | 123 (67.21) |
| Family history of breast cancer |  |  |  |
| No | 674 (82.90) | 308 (76.43) | 142 (79.78) |
| Yes | 139 (17.10) | 95 (23.57) | 36 (20.22) |
| Body mass index (BMI) |  |  |  |
| BMI<25kg/m^2^ | 397 (48.06) | 156 (37.50) | 74 (40.00) |
| BMI 25-29.9 kg/m^2^ | 269 (32.57) | 137 (32.93) | 55 (29.73) |
| BMI≥30kg/m^2^ | 160 (19.37) | 123 (29.57) | 56 (30.27) |
| Cigarette Smoking |  |  |  |
| Never | 380 (45.62) | 188 (44.66) | 90 (48.39) |
| Current/Former | 453 (54.38) | 233 (55.34) | 96 (51.61) |
| History of benign breast disease |  |  |  |
| No | 655 (78.83) | 356 (84.56) | 150 (80.65) |
| Yes | 177 (21.27) | 65 (15.44) | 36 (19.35) |
| Cancer type |  |  |  |
| in situ | 164 (19.69) | 29 (6.89) | 5 (2.69) |
| invasive | 669 (80.31) | 392 (93.11) | 181 (97.31) |
| Hormone receptor status^a^ |  |  |  |
| positive | 435 (81.77) | 235 (75.56) | 105 (71.43) |
| negative | 97 (18.23) | 76 (24.44) | 42 (28.57) |
| Estrogen receptor status |  |  |  |
| positive | 410 (77.07) | 220 (70.74) | 95 (64.63) |
| negative | 122 (22.93) | 91 (29.26) | 52 (35.37) |
| Progesterone receptor status |  |  |  |
| positive | 370 (69.55) | 178 (57.23) | 80 (54.42) |
| negative | 162 (30.45) | 133 (42.77) | 67 (45.58) |
| Tumor size |  |  |  |
| <2cm | 318 (70.04) | 134 (57.76) | 47 (42.34) |
| ≥2cm | 136 (29.96) | 98 (42.24) | 64 (57.66) |
| Nodal involvement |  |  |  |
| 0 | 108 (23.84) | 58 (24.37) | 29 (25.00) |
| 1 | 345 (76.16) | 180 (75.63) | 87 (75.00) |
| Treatment type |  |  |  |
| no chemotherapy | 378 (61.46) | 131 (55.27) | 35 (33.02) |
| chemotherapy | 237 (38.54) | 106 (44.73) | 71 (66.98) |
|  |  |  |  |
| no radiation | 245 (39.84) | 91 (37.92) | 42 (39.25) |
| radiation | 370 (60.16) | 149 (62.08) | 65 (60.75) |
|  |  |  |  |
| no hormone therapy | 245 (40.03) | 76 (33.19) | 27 (26.73) |
| hormone therapy | 367 (59.97) | 153 (66.81) | 74 (73.27) |
| ^a^ Any ER-positive or PR-positive tumor | |  |  |
