# Supplementary material for: Modification of the association between recreational physical activity and survival after breast cancer by promoter methylation in breast cancer-related genes
Source: Breast Cancer Res. 2017 Feb 21;19:19. doi: 10.1186/s13058-017-0811-z (PMC5319077; doi:10.1186/s13058-017-0811-z)
Supplement: Additional file 2: Table S2. — Gene function and previously observed methylation status of the 13 investigated genes in relation to recreational physical activity (RPA) and breast cancer-specific mortality, Long Island Breast Cancer Study Project. (DOC 36 kb) [file 13058_2017_811_MOESM2_ESM.doc]

| **Additional file 2: Table S2.** Gene function and previously observed methylation status of the 13 investigated genes in relation to RPA and breast cancer-specific mortality, Long Island Breast Cancer Study Project. | | | | |
| --- | --- | --- | --- | --- |
| Gene | Group^a^ | Function^a^ | Previously Reported Association with Breast Cancer^b^ | Previously Reported Association with RPA^c^ |
| ESR1 | steroid hormone receptor | hormone binding, DNA binding and activation of transcription | 1.13 (0.80, 1.60) | 0.73 (0.46, 1.15) |
| PGR | steroid hormone receptor | mediates the physiological effects of progesterone | 0.70 (0.30, 1.63) | 0.95 (0.48, 1.89) |
| BRCA1 | tumor suppressor | maintaining genomic stability | 1.78 (1.22, 2.62) | 1.04 (0.66, 1.64) |
| APC | tumor suppressor | an antagonist of the Wnt signaling; Defects cause familial adenomatous polyposis | 1.53 (1.07, 2.20) | 1.00 (0.62, 1.60) |
| p16/CDKN2A | tumor suppressor | cell cycle control | 2.28 (1.19, 4.35) | 0.52 (0.17, 1.59) |
| HIN1/SCGB3A1 | tumor suppressor | growth-inhibitory cytokine, regulates epithelial cell differentiation | 1.18 (0.80, 1.74) | 1.44 (0.87, 2.38) |
| RASSF1A | tumor suppressor | involved in cell cycle control | 1.42 (0.80, 2.53) | 0.79 (0.41, 1.53) |
| DAPK1 | tumor suppressor | positive mediator of gamma- interferon induced programmed cell death | 1.25 (0.77, 2.04) | 1.21 (0.64, 2.29) |
| GSTP1 | detoxification | xenobiotic metabolism | 1.85 (1.27, 2.71) | 0.90 (0.53, 1.54) |
| CCND2 | oncogene | regulators of CDK kinases | 1.27 (0.89, 1.99) | 0.81 (0.45, 1.46) |
| TWIST1 | transcription factors | cell lineage determination and differentiation | 1.58, 1.00, 2.50) | 0.94 (0.49, 1.80) |
| CDH1 | tumor suppressor | cell proliferation, invasion, and/or metastasis | 1.22 (0.57, 2.63) | 0.37 (0.11, 1.19) |
| RARβ | steroid hormone receptor | mediates cellular signaling in embryonic morphogenesis, cell growth and differentiation | 1.50 (1.02, 2.20) | 0.62 (0.36, 1.06) |
| ^a^Previously summarized by Xu et al. 2011 [31]. ^b^Previously reported by McCullough et al. 2016 [33] with follow-up through 2011. ^c^Previously reported by McCullough et al. 2015 [32]. | | | | |
