# Supplementary material for: Modification of the association between recreational physical activity and survival after breast cancer by promoter methylation in breast cancer-related genes
Source: Breast Cancer Res. 2017 Feb 21;19:19. doi: 10.1186/s13058-017-0811-z (PMC5319077; doi:10.1186/s13058-017-0811-z)
Supplement: Additional file 3: Table S3. — Age-adjusted HRs and 95% CIs for the association between lifetime recreational physical activity (RPA) and 15-year all-cause and breast cancer-specific mortality stratified by gene methylation status (methylated vs. unmethylated tumors) among 803 women diagnosed with a first primary hormone receptor-positive breast cancer and with available gene promoter methylation data, Long Island Breast Cancer Study Project. (DOC 56 kb) [file 13058_2017_811_MOESM3_ESM.doc]

| **Additional file 3: Table S3.** Age-adjusted hazard ratios (HRs) and 95% confidence intervals (CIs) for the association between lifetime recreational physical activity (RPA) and 15-year all-cause and breast cancer-specific mortality stratified by gene methylation status (methylated vs. unmethylated tumors) among 803 women diagnosed with a first primary hormone receptor positive breast cancer and with available gene promoter methylation data, Long Island Breast Cancer Study Project. | | | | | | | | | | | | |
| --- | --- | --- | --- | --- | --- | --- | --- | --- | --- | --- | --- | --- |
|  | **All Cause Mortality** | | | | | | **Breast Cancer Specific Mortality** | | | | | |
|  | Unmethylated | | | Methylated | | | Unmethylated | | | Methylated | | |
| ***Gene promoter***  RPA categories | No. deaths/ cases | HR | 95% CI | No. deaths/ cases | HR | 95% CI | No. deaths/ cases | HR | 95% CI | No. deaths/ cases | HR | 95% CI |
| ***APC*** |  |  |  |  |  |  |  |  |  |  |  |  |
| Inactive | 21/46 | 1.00 | reference | 29/56 | 1.00 | reference | 7/46 | 1.00 | reference | 15/56 | 1.00 | reference |
| <6.36 hrs/wk | 33/106 | 0.83 | (0.47, 1.46) | 21/72 | 0.45 | (0.26, 0.79) | 12/106 | 0.54 | (0.21, 1.40) | 5/72 | 0.21 | (0.08, 0.58) |
| ≥6.36 hrs/wk | 25/75 | 0.80 | (0.45, 1.44) | 34/89 | 0.68 | (0.41, 1.11) | 11/75 | 0.74 | (0.29, 1.93) | 20/89 | 0.70 | (0.36, 1.36) |
| *p interaction* | 0.090 | | | | | | 0.082 | | | | | |
| ***CYCLIND2*** |  |  |  |  |  |  |  |  |  |  |  |  |
| Inactive | 29/76 | 1.00 | reference | 18/26 | 1.00 | reference | 10/76 | 1.00 | reference | 9/26 | 1.00 | reference |
| <6.36 hrs/wk | 43/140 | 0.91 | (0.56, 1.46) | 11/29 | 0.36 | (0.17, 0.75) | 17/140 | 0.77 | (0.35, 1.70) | <5/29 | not estimated^a^ | |
| ≥6.36 hrs/wk | 37/115 | 0.92 | (0.56, 1.50) | 20/40 | 0.59 | (0.31, 1.12) | 18/115 | 1.03 | (0.47, 2.25) | 9/40 | 0.48 | (0.19, 1.22) |
| *p interaction* | 0.033 | | | | | | not estimated | | | | | |
| ***HIN*** |  |  |  |  |  |  |  |  |  |  |  |  |
| Inactive | 10/37 | 1.00 | reference | 37/65 | 1.00 | reference | 3/37 | 1.00 | reference | 16/65 | 1.00 | reference |
| <6.36 hrs/wk | 18/62 | 1.15 | (0.53, 2.51) | 36/107 | 0.57 | (0.36, 0.90) | 5/62 | 0.82 | (0.19, 3.54) | 13/107 | 0.36 | (0.17, 0.75) |
| ≥6.36 hrs/wk | 14/65 | 1.39 | (0.61, 3.17) | 43/110 | 0.63 | (0.41, 0.98) | 5/65 | 1.28 | (0.30, 5.41) | 22/110 | 0.60 | (0.32, 1.15) |
| *p interaction* | 0.052 | | | | | | 0.172 | | | | | |
| ***TWIST1*** |  |  |  |  |  |  |  |  |  |  |  |  |
| Inactive | 32/86 | 1.00 | reference | 15/16 | 1.00 | reference | 13/86 | 1.00 | reference | 6/16 | 1.00 | reference |
| <6.36 hrs/wk | 45/144 | 0.92 | (0.58, 1.45) | 9/25 | 0.20 | (0.08, 0.48) | 16/144 | 0.68 | (0.33, 1.43) | <5/25 | not estimated | |
| ≥6.36 hrs/wk | 48/129 | 1.13 | (0.72, 1.77) | 9/26 | 0.21 | (0.09, 0.50) | 20/129 | 1.04 | (0.52, 2.11) | 7/26 | 0.16 | (0.05, 0.50) |
| *p interaction* | 0.001 | | | | | | not estimated | | | | | |
| ^a^ Point estimate was not calculated because cell sizes less than five | | | | |  |  |  |  |  |  |  |  |
