# Supplementary material for: Modification of the association between recreational physical activity and survival after breast cancer by promoter methylation in breast cancer-related genes
Source: Breast Cancer Res. 2017 Feb 21;19:19. doi: 10.1186/s13058-017-0811-z (PMC5319077; doi:10.1186/s13058-017-0811-z)
Supplement: Additional file 4: Table S4. — Age-adjusted HRs and 95% CIs for the association between lifetime recreational physical activity (RPA) and 15-year all-cause and breast cancer-specific mortality, stratified by global methylation status (measured by LUMA and LINE-1), among a population-based sample of 1015 women diagnosed with a first primary breast cancer and with available global methylation data, Long Island Breast Cancer Study Project. (DOC 43 kb) [file 13058_2017_811_MOESM4_ESM.doc]

| **Additional file 4: Table S4.** Age-adjusted hazard ratios (HRs) and 95% confidence intervals (CIs) for the association between lifetime recreational physical activity (RPA) and 15-year all-cause and breast cancer-specific mortality, stratified by global methylation status (measured by LUMA and LINE-1), among a population-based sample of 1015 women diagnosed with a first primary breast cancer and with available global methylation data, Long Island Breast Cancer Study Project. | | | | | | | | | | | | |
| --- | --- | --- | --- | --- | --- | --- | --- | --- | --- | --- | --- | --- |
|  | **All-Cause Mortality** | | | | | | **Breast Cancer-Specific Mortality** | | | | | |
| **Global marker** RPA categories | No. deaths/ cases | HR | 95% CI | No. deaths/ cases | HR | 95% CI | No. deaths/ cases | HR | 95% CI | No. deaths/ cases | HR | 95% CI |
| **LUMA methylation^a^** | < Median | | | ≥ Median | | | < Median | | | ≥ Median | | |
| Inactive | 38/80 | 1.00 | reference | 63/150 | 1.00 | reference | 18/80 | 1.00 | reference | 28/150 | 1.00 | reference |
| <6.36 hrs/wk | 45/150 | 0.76 | (0.49, 1.18) | 67/252 | 0.67 | (0.47, 0.95) | 23/150 | 0.59 | (0.31, 1.12) | 32/252 | 0.58 | (0.35, 0.97) |
| ≥6.36 hrs/wk | 37/123 | 0.65 | (0.42, 1.03) | 77/250 | 0.71 | (0.51, 0.99) | 15/123 | 0.48 | (0.24, 0.96) | 29/250 | 0.56 | (0.33, 0.94) |
| *p interaction* | 0.493 | | | | | | 0.654 | | | | | |
|  |  |  |  |  |  |  |  |  |  |  |  |  |
| **LINE-1 methylation^b^** | ≥ Median | | | < Median | | | ≥ Median | | | < Median | | |
| Inactive | 48/111 | 1.00 | reference | 53/122 | 1.00 | reference | 21/111 | 1.00 | reference | 25/122 | 1.00 | reference |
| <6.36 hrs/wk | 51/189 | 0.75 | (0.50, 1.12) | 63/217 | 0.68 | (0.47, 0.99) | 23/189 | 0.54 | (0.30, 0.99) | 33/217 | 0.63 | (0.38, 1.07) |
| ≥6.36 hrs/wk | 56/190 | 0.75 | (0.51, 1.11) | 59/186 | 0.66 | (0.45, 0.95) | 22/190 | 0.50 | (0.27, 0.92) | 23/186 | 0.53 | (0.30, 0.94) |
| *p interaction* | 0.609 | | | | | | 0.668 | | | | | |
| ^a^ LUMA methylation median value 0.556, high levels of LUMA hypothesized to be deleterious | | | | | | | |  |  |  |  |  |
| ^b^ LINE-1 methylation median value 78.735, low levels of LINE-1 hypothesized to be deleterious | | | | | | | |  |  |  |  |  |
